# Efficient System for Delimitation of Benign and Malignant Breast Masses

**DOI:** 10.3390/e24121775

**Published:** 2022-12-05

**Authors:** Dante Mújica-Vargas, Manuel Matuz-Cruz, Christian García-Aquino, Celia Ramos-Palencia

**Affiliations:** 1 Departamento de Ciencias Computacionales, Tecnológico Nacional de México, Centro Nacional de Investigación y Desarrollo Tecnológico, Cuernavaca 62490, Morelos, Mexico; 2 Tecnológico Nacional de México, Instituto Tecnológico de Tapachula, Tapachula 30700, Chiapas, Mexico

**Keywords:** breast masses delimitation, benign and malignant breast tumors, ultrasound image, nonlocal means, intuitionistic fuzzy clustering, DBSCAN

## Abstract

In this study, a high-performing scheme is introduced to delimit benign and malignant masses in breast ultrasound images. The proposal is built upon by the Nonlocal Means filter for image quality improvement, an Intuitionistic Fuzzy C-Means local clustering algorithm for superpixel generation with high adherence to the edges, and the DBSCAN algorithm for the global clustering of those superpixels in order to delimit masses’ regions. The empirical study was performed using two datasets, both with benign and malignant breast tumors. The quantitative results with respect to the BUSI dataset were JSC≥0.907, DM≥0.913, HD≥7.025, and MCR≤6.431 for benign masses and JSC≥0.897, DM≥0.900, HD≥8.666, and MCR≤8.016 for malignant ones, while the MID dataset resulted in JSC≥0.890, DM≥0.905, HD≥8.370, and MCR≤7.241 along with JSC≥0.881, DM≥0.898, HD≥8.865, and MCR≤7.808 for benign and malignant masses, respectively. These numerical results revealed that our proposal outperformed all the evaluated comparative state-of-the-art methods in mass delimitation. This is confirmed by the visual results since the segmented regions had a better edge delimitation.

## 1. Introduction

One of the most commoninvasive cancers in women worldwide is breast cancer [1]. In many cases, early detection accompanied with appropriate treatments may significantly reduce the need for surgery and increase women’s survival rate. At present, breast cancer medical imaging is performed by using the follow methods: X-ray mammography, breast ultrasound, Magnetic Resonance Imaging (MRI) [2], and the new 3D tomography technique called Digital Breast Tomosynthesis (DBT) [3]. Breast image analyzing is a difficult task that radiologists must perform frequently, which consists of detection, recognition, segmentation, and classification tasks. To boost the radiologist effectiveness, computer-assisted analysis techniques based on digital image processing must be used to improve the visual quality and detect any abnormalities that may exist to assist the radiologist’s labor. These abnormalities are conventionally referred to as breast masses in the literature and are classified as benign or malignant [4]. In general, benign masses are well-defined compared to malignant ones. Therefore, in the context of breast mass delimitation, processing malignant tumors may be considered more difficult than processing benign ones. As a significant contribution to this research area, a system capable of efficiently dealing with both breast mass types is proposed, that is, a proposal that is not limited to a particular case of abnormalities.

Taking the relevance of breast cancer into account, researchers have attempted to develop computer-aided systems and have introduced different computational algorithms to help in clinical practice. The state-of-the-art evidence shows that in recent times, there has been a clear trend towards Deep Learning. This paradigm offers a spectrum of architectures, such as Convolutional Neural Networks, that may offer solutions for a range of problem areas, such as that in the current study. In this respect, the U-Net architecture and its variants are the most commonly used for breast mass segmentation. For instance, in [3], an approach that consisted of six main stages—DBT image preprocessing, patch extraction, data augmentation, voting scheme fusion, segmentation via the U-Net architecture, and postprocessing—was proposed. The experimental evaluation of this proposal achieved a performance of Acc=0.871, Sen=0.869, Spe=0.882, and AUC=0.859. In [5], the introduced proposal was based on selective kernels (SKs), which automatically adjusted the network’s receptive field via attention mechanisms and mix feature maps extracted with both dilated and conventional convolutions. With this modification to the U-Net architecture, a mean Dice score of 0.826 and Spearman rank coefficient of 0.7 were achieved. In [6], the authors proposed a transformation of ultrasound images to entropy parametric maps and then used them to train a U-Net. This proposal achieved an average Dice score of 0.60. In [7], ten U-Net models were evaluated with different settings under ten-fold cross-validation, and the final segmentation was chosen by majority voting. The proposal achieved a mean Dice Coefficient of 0.82, a true-positive fraction of 0.84, and a false positive-fraction of 0.01. In [8], the introduced procedure enhanced the images using the Contrast Limited Adaptive Histogram Equalization method, and then it reduced the noise via the Bilateral Filter. The masses were segmented with a U-Net modified with VE blocks, each of which consisted of a concatenated max and average pooling with batch normalization. The results indicated that the proposed method achieved a Dice Measure of 89.73 for malignant masses and 89.62 for benign masses. In [9], the contracting path of a U-Net architecture was replaced by five feature stages following the ResNet-34 architecture in order to incorporate features with different resolutions. This proposal obtained a Dice Coefficient of 0.887 and an Intersection over Union of 0.804. In [10], a procedure to segment the breast mass in 3D images was proposed, and the scheme was equipped with the $D^{2}$U-Net scheme for a better multi-scale feature extraction. It also incorporated dilated convolutions into the densely connected block in a hybrid manner, which enabled the network to handle both large and small objects without increasing the network parameters. In [11], attention blocks were introduced to a U-Net architecture, with the aim of learning feature representations that prioritize spatial regions with high salience levels. The approach achieved a Dice Similarity Coefficient of 0.905.

There are also proposals based on a Region-Based Convolutional Neural Network (RCNN) in conjunction with other architectures. In [12], magnetic resonance images were improved via a dynamic contrast-enhanced procedure. In addition, the regions of the breasts were segmented from the remaining body parts by building a fully convolutional neural network based on U-Net++. Then, a Faster RCNN was used for mass detection on segmented breast images. In [13], the introduced proposal was focused on tasks such as the region of interest detection and lesion localization, which may be considered as a primitive tumor delimitation. In [14], a target detection model known as D-Mask R-CNN was proposed and based on Mask R-CNN. The model used the dense connection method in DenseNet to replace the lateral connection method of Feature Pyramid Network in the original network. The experimental results showed an Average Precision of 0.78 and an Intersection over Union of 0.75. In [15], the Densenet264 model was optimized by adding a learning scalable feature pyramid architecture, training at multiple scales, and adding the ResNet-C input stem. The performance obtained a Dice score of 0.832, a Cohen’s kappa of 0.823, and a Sensitivity of 0.826. In [16], the ultrasonic breast lesion images were segmented using a Dilated Semantic Segmentation Network combined with a morphological erosion operation, and the proposal also included a DenseNet 201 model in the classification stage. In [17], a two-stage multi-scale pipeline was proposed for mass localization and segmentation from high-resolution X-ray mammograms. It consisted of a YOLOv3 model for detection and a V19U-Net++ for segmentation and was able to achieve a Dice score of 80.44. In [18], an approach that incorporated a Bidirectional Long Short-Term Memory and Spatial-Channel Attention module into a Fully Convolutional Network was proposed. This proposal obtained a Dice score of 0.8178 and a Hausdorff Distance of 11.136, among other metrics.

On the other hand, conventional proposals are still being introduced to the state of the art, highlighting those based on clustering. For instance, [19] introduced multiple loss functions designed to aid in clustering image pixels that were spatially connected and had similar feature representations, which iteratively learned feature representations and clustering assignment of each pixel in an end-to-end fashion from a single image; the proposal achieved a Dice score of 0.843. In [20], a three-layer system was introduced, and initially, the noise was reduced by the Anisotropic Diffusion filter, then a Multi-scale Gaussian-Kernel-induced Fuzzy C-means was used for tumor segmentation, and finally, a Multi-scale Vector Filed Convolution was utilized to determine the accurate lesion margin; this approach was evaluated by taking into account just five image samples.

In [21], a scheme is introduced that consisted of nine processes. The pipeline included Crop ROI, bilateral filtering, histogram equalization, mean shift filtering, generating superpixels using SLIC, extracting features (gray histogram, GLCM and co-occurrence matching of LBP), Kmeans and a bag-of-words model, BPNN classification for initial results, and KNN for reclassification and postprocessing. The scheme was tested with 100 breast ultrasound imagesl it had an F1-score of 89.87±4.05. In [22], an automatic two-phase method was proposed; the first phase aimed to detect the tumor and obtain an initial tumor outline by means of SLIC, and the second was focused on improving the accuracy of the initial tumor outline via a customized Graph Cuts algorithm. In [23], a Spatial Fuzzy C-Means was used to segment masses on a Dynamic Contrast-Enhanced MRI of the breast; it obtained a Dice similarity coefficient of 84.47±4.75. In addition, there are approaches based on more classical techniques, such as the proposal introduced in [24], where a strategy for initialization of active contours was introduced, which consisted of a proposed fusion method and a radial force based on the fusion of the conventional US, Doppler, and elasticity images; its efficiency implied a Jaccard Similarity Coefficient of 0.89 and a Dice Measure of 0.9. Finally, Ref. [25] proposed an automatic segmentation approach that extracted features from dynamic contrast-enhanced and diffusion-weighted MRI; it achieved a delimitation of an Average Accuracy of 0.87.

Reference [26], an interesting study that was carried out, was about a comparison between Deep Learning and Superpixels. According to the authors, Deep Learning Neural Networks were able to realize accurate segmentation; in contrast, the Superpixels retained the mass boundary information to a large extent. Both results are also important for breast masses’ segmentation; consequently, even if Deep Learning is a powerful paradigm, the virtues of traditional methods are still required. In support of the last contention, in this study, a processing scheme composed of three algorithms with high performance is introduced. It is based on algorithms such as Nonlocal Means filter [27], Intuitionistic Fuzzy C-Means [28], and Density-Based Spatial Clustering of Applications with the Noise algorithm, commonly known as DBSCAN [29]. Each of these algorithms has a particular role: Nonlocal Means filter improves image quality by reducing existing noise while the edges are preserved; the Intuitionistic Fuzzy C-Means algorithm performs the superpixel extraction with high edge adherence, and the DBSCAN approach clusters the superpixels in order to segment the breast masses. The contributions of this study are summarized as follows:

1. A competent system capable of delimiting breast benign and malignant masses, which consists of a simple pipeline processing, with a denoising stage, as well as local and global clustering procedures.
2. A system with a reduced number of internal parameters, most of them required for the Intuitionistic Fuzzy stage. Specifically, they are transformation factor λ, fuzzifier factor γ, and the superpixels number *K*, which can be set to a specific value (see Section 2.2). It is an important advantage over all methods mentioned in the state-of-the-art review.
3. Superpixels are generated and processed in the Intuitionistic Fuzzy domain, which has an advantage compared to superpixels processed in the crisp domain (by SLIC), since a better adhesion to the edges can be guaranteed.
4. The DBSCAN is adjusted in order to cluster superpixels instead conventional pixels.
5. The system does not require processing special features, since it works only with the pixels’ intensity.

The rest of this paper is organized as follows. In Section 2, the mathematical background of each used algorithm is described in detail. In Section 3, the proposed scheme is described in brief. Experimental results and a comparative analysis with other current methods in the literature are presented in Section 4. The concluding section gives a synopsis of the principal results and recommendations for future work.

## 2. Materials and Methods

### 2.1. Ultrasound Image Denoising

Noise is inherently present in ultrasound images; its generation source may include the equipment used for acquisition to the presence of different organs and tissues. The noise existing in ultrasound images is modeled as non-Gaussian and multiplicative, usually referred to as speckles [30]; therefore, this noise is called speckle noise. Processing ultrasound images is a very challenging task due to this speckle noise. Thus, in this study, it is considered an edge-preserving smoothing filter to improve the quality of the image, specifically the Nonlocal Means filter. The edge preserving is achieved when the filter changes its smoothing behavior adaptively depending on the local image structure.

Let a noisy image $\overset{ˇ}{X}=\left\{\overset{ˇ}{x}\left(i\right)|i\in I\right\}$; the estimation for the *i*-th pixel is computed by weighted averaging of all pixels with similar intensity. Mathematically, the Nonlocal Means filter is expressed as [27]:

(1) $$NL\left(\overset{ˇ}{x}\right)\left(i\right)=\sum_{j\in I}w\left(i,j\right)\overset{ˇ}{x}\left(j\right),$$

where w(i,j) quantifies the affinity between the *i*-th and *j*-th pixels, taking into account the 0≤w(i,j)≤1 and $\sum_{j\in I}w\left(i,j\right)=1$ constraints. The gray intensity vectors $\overset{ˇ}{x}\left(N_{i}\right)$ and $\overset{ˇ}{x}\left(N_{j}\right)$ define the relationship between *i* and *j* pixels, where $N_{k}$ is the square neighborhood of fixed size and *k* is the center pixel. The pixels with a similar gray-level neighborhood to $\overset{ˇ}{x}\left(N_{i}\right)$ have larger weights than the average. These weights are calculated as [31]:

(2) $$w\left(i,j\right)=\frac{1}{Z_{i}}\exp^{-\frac{\|\overset{ˇ}{x}\left(N_{i}\right)-\overset{ˇ}{x}\left(N_{j}\right){\|}_{2}^{2}}{h^{2}}},$$

(3) $$Z_{i}=\exp^{-\frac{\|\overset{ˇ}{x}\left(N_{i}\right)-\overset{ˇ}{x}\left(N_{j}\right){\|}_{2}^{2}}{h^{2}}},$$

where $N_{i}$ and $N_{j}$ are intensities of local neighborhood centers on pixels *i* and *j*, $Z_{i}$ stands for a normalization constant, and *h* is the degree of filtering that controls the decay of the exponential function. For convenience, the filtered image $NL(\overset{ˇ}{x})$ will be referred to simply as *X*.

### 2.2. Grayscale Image Oversegmentation Using Intuitionistic Fuzzy Theory

Let us consider a grayscale breast ultrasound image $X=\{x_{mn}|m=1,\ldots ,M;n=1,\ldots ,N\}$ with *M* rows and *N* columns, and its vector form $\vec{X}=\left\{{\vec{x}}_{l}|l=1,\ldots ,L\right\}$, where L=M×N. For processing this image into an Intuitionistic Fuzzy domain, it must be transformed using:

(4) $$x_{l}^{IFS}=\left\{\langle {\vec{x}}_{l},\mu \left({\vec{x}}_{l}\right),\nu \left({\vec{x}}_{l}\right),\pi \left({\vec{x}}_{l}\right)\rangle \forall \;\vec{x_{l}}\in \vec{X}|l=1,\ldots L\right\},$$

where $\mu \left({\vec{x}}_{l}\right),\nu \left({\vec{x}}_{l}\right)$ and $\pi ({\vec{x}}_{l})$ are the membership, non-membership, and hesitancy degrees, respectively. Expression (1) states that every pixel is treated as an individual intuitionistic fuzzy set constrained by $\mu \left({\vec{x}}_{l}\right)+\nu \left({\vec{x}}_{l}\right)+\pi \left({\vec{x}}_{l}\right)=1$ and $0\leq \pi ({\vec{x}}_{l})\leq 1$. These three indices are computed as:

(5) $$\mu \left({\vec{x}}_{l}\right)=\frac{{\vec{x}}_{l}-\min\left(\vec{X}\right)}{\max\left(\vec{X}\right)-\min\left(\vec{X}\right)},$$

(6) $$\nu \left({\vec{x}}_{l}\right)=\frac{1-\mu ({\vec{x}}_{l})}{1+\left(e^{\lambda }-1\right)\;\cdot \;\mu \left({\vec{x}}_{l}\right)},\;\lambda \in \left[0,1\right],$$

(7) $$\pi \left({\vec{x}}_{l}\right)=1-\mu \left({\vec{x}}_{l}\right)-\nu \left({\vec{x}}_{l}\right),$$

where the $\min(\vec{X})$ and $\max(\vec{X})$ functions calculate the minimum and maximum value of $\vec{X}$, respectively. In this study, λ is a parameter defined by the user.

The image segmentation may be performed as a global clustering process; in contrast, the oversegmentation implies a local clustering procedure, by convention the local clusters will be called superpixels. Taking into account that remark, next objective function may be stated as the next functional:

(8) $$J_{m}\left({\vec{X}}^{IFS};U,V^{IFS}\right)=\sum_{l=1}^{L}\sum_{k=1}^{K}u_{lk}^{\gamma }{∥{\vec{x}}_{l}^{IFS}-v_{k}^{IFS}∥}_{2}^{2}.$$

The input ${\vec{X}}^{IFS}=\left\{{\vec{x}}_{l}^{IFS}|l=1,\ldots ,L\right\}$ is a vector with *l* intuitionistic fuzzy sets. $V^{IFS}=\left\{v_{k}^{IFS}|k=1,\ldots ,K\right\}$ is a vector with the *K* initial superpixels centroids; in this study, it is suggested that K=1000, $v_{k}^{IFS}$ is a tuple given by $v_{k}^{IFS}=\left\{\mu \left({\vec{x}}_{k}\right),\nu \left({\vec{x}}_{k}\right),\pi \left({\vec{x}}_{k}\right)\right\}$. Additionally, $U=\{u_{lk}|l=1,\ldots ,L;\;k=1,\ldots ,K\}$ is the cluster partition of ${\vec{X}}^{IFS}$; where, $u_{lk}$ quantifies the pixel membership ${\vec{x}}_{l}^{IFS}$ to the *k*-th superpixel. A fuzzifier parameter γ≥2 is suggested. Lastly, the square Euclidean intuitionistic fuzzy distance ${∥\;\cdot \;∥}_{2}^{2}$ may be computed as:

(9) $$\begin{array}{c} \begin{array}{c} ∥{\vec{x}}_{l}^{IFS}-v_{k}^{IFS}{∥}_{2}^{2}=\left\{{\left(\mu \left({\vec{x}}_{l}\right)-\mu \left({\vec{x}}_{k}\right)\right)}^{2}+{\left(\nu \left({\vec{x}}_{l}\right)-\nu \left({\vec{x}}_{k}\right)\right)}^{2}+{\left(\pi \left({\vec{x}}_{l}\right)-\pi \left({\vec{x}}_{k}\right)\right)}^{2}\right\}. \end{array} \end{array}$$

The updated expressions for internal variables are obtained by setting the partial derivative of $J_{m}$ with respect to the optimization parameters equal to zero. Regarding the lk-th membership degree, following updated equation may be considered:

(10) $$u_{lk}=\frac{1}{\sum_{r=1}^{K}{\left[\frac{∥{\vec{x}}_{l}^{IFS}-v_{k}^{IFS}{∥}_{2}^{2}}{∥{\vec{x}}_{l}^{IFS}-v_{r}^{IFS}{∥}_{2}^{2}}\right]}^{\frac{1}{m-1}}}\;.$$

The prototypes vector is computed by:

(11) $$v_{k}^{IFS}=\left\{\frac{\sum_{l=1}^{L}u_{lk}^{\gamma }\mu \left({\vec{x}}_{k}\right)}{\sum_{l=1}^{L}u_{lk}^{\gamma }},\frac{\sum_{l=1}^{L}u_{lk}^{\gamma }\nu \left({\vec{x}}_{k}\right)}{\sum_{l=1}^{L}u_{lk}^{\gamma }},\frac{\sum_{l=1}^{L}u_{lk}^{\gamma }\pi \left({\vec{x}}_{k}\right)}{\sum_{l=1}^{L}u_{lk}^{\gamma }}\right\}\;.$$

**Figure 1:**
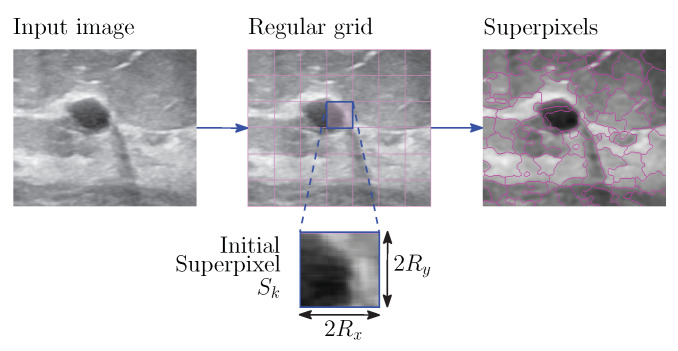
Initial grids.

For the current study, local clustering means that a segmentation process must be developed on each *k*-th superpixel by using Expressions (8), (10) and (11). To that end, the input image is partitioned into regular grids (see Figure 1) with a 2R×2R size, where $R=\sqrt{L/K}$. The constraint $X={⋃}_{k=1}^{K}S_{k}$ must be respected, where $S_{k}$ is the *k*-th superpixel.

### 2.3. Clustering of Intuitionistic Fuzzy Superpixels

**Figure 2:**
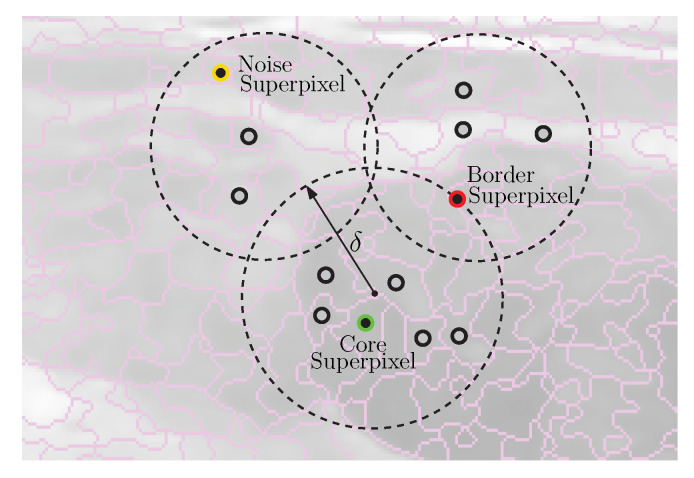
Representation of superpixel centroids and the neighborhood radius.

Figure 1 depicts how superpixels are adjusted to small region edges; however, the purpose in this paper implies delimiting the existing breast masses; therefore, a clustering process of the intuitionistic fuzzy superpixels must be performed. This task is developed by the DBSCAN algorithm. Unlike most clustering algorithms, DBSCAN uses local connectivity and density functions to perform the clustering procedure, which it is a advantage because it does not require cluster initialization [32]. For the use of DBSCAN in the present study, it should be assumed that every superpixel is described by its centroid, as shown in Figure 2.

In addition, this figure depicts other parameters, for instance, the *Core Superpixels*, which are superpixels that are within the δ radius and have at least ϑ neighbors; the *Border Superpixels*, which are superpixels on the border, as well as the *Noise Superpixels*, which are superpixels that do not correspond to any of the previous types. DBSCAN is stated by the maximum radius of the neighborhood δ and the minimum number of superpixels in the neighborhood ϑ bounded by δ. In essence, the algorithm is a heuristic that analyzes and clusters the superpixels in terms of γ distance. This means that the algorithm includes all core superpixels that are within γ distance of each other in the same cluster and excludes border and noise superpixels of the interest clusters.

## 3. Proposed Scheme

**Figure 3:**
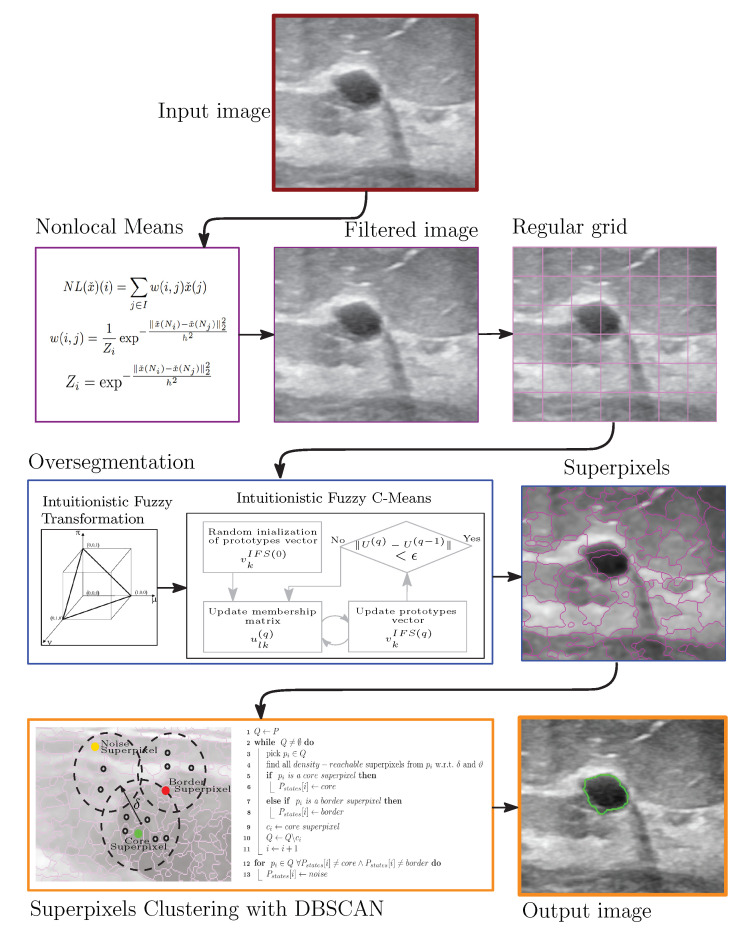
Proposed system.

Figure 3 shows the proposed approach for detecting and breast masses’ delimitation; it basically consists of three stages. First, the image is subject to a preprocessing task by the powerful Nonlocal Means filter; by means of Expressions (1)–(3), the multiplicative noise is reduced while the edges are preserved. After that, the image is oversegmented in order to generate the superpixels. In this respect, the image must be divided into a regular grid, and then every local region is transformed into Intuitionistic Fuzzy domain via Expressions (4)–(7). Subsequently the local over clustering is developing by using Equations (8)–(11). At a later stage, the DBSCAN algorithm develops the superpixel clustering for delimiting the edges of breast masses; to do so, all steps suggested in Algorithm 1 must be followed.
**Algorithm 1:** Intuitionistic Fuzzy Superpixels-DBSCAN
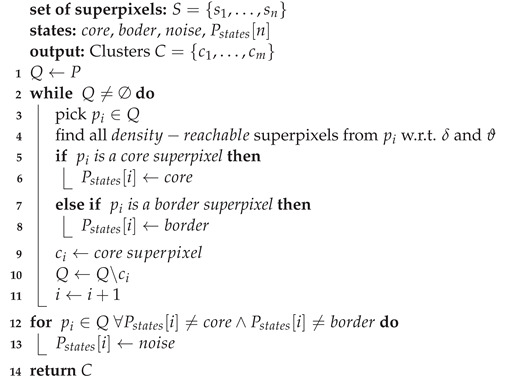


## 4. Experimental Results

Two public datasets were considered in this study, Breast Ultrasound Images Dataset (BUSI) [33] and Medical Image Database (MID) [34]. Both datasets include healthy, benign, malignant and other sample types. In particular, 421 benign and 891 malignant samples were taken from the BUSI dataset, while 76 benign and 217 malignant samples were taken from the MID dataset.

For both databases, next, comparative algorithms and schemes were replicated: Salient Attention Contour U-Net (SAC U-Net), Spatial Guided Self Supervised Clustering (SGSCN), and Automatic Superpixel-based Segmentation Method (ASbSM). For convenience, the proposed system will reference NL-IFS-DBSCAN too allude to the three base algorithms. All evaluated approaches were programmed in Python or MATLAB, using an Intel® Core™ processor i7-4720HQ CPU @2.60 GHz with four cores and 16 GB of RAM memory.

### 4.1. Metrics

The quantitative performance of the evaluated methods is carried out through well-known metrics from the literature, such as Jaccard Similarity Coefficient (JSC) [8], Dice Measure (DM) [8], and Hausdorff Distance (HD) [8], as well as Misclassification Ratio (MCR) [35]. They are computed by the following equations:

(12) $$JSC\left(X,Y\right)=\frac{|X\cap Y|}{|X\cup Y|},$$

(13) $$DM\left(X,Y\right)=2\;\cdot \;\frac{|X\cap Y|}{|X|+|Y|},$$

(14) $$\begin{array}{c} \begin{array}{c} HD\left(X,Y\right)=\max\left\{{\max(\min(\|X-Y\|}_{2}{)),\max(\min(\|Y-X\|}_{2}))\right\}, \end{array} \end{array}$$

(15) $$MCR=\frac{misclassified\;pixels}{overall\;number\;of\;pixels}\times 100,$$

where *X* is the segmented image and *Y* is the ground truth.

### 4.2. Experiment 1: BUSI Dataset

Table 1 summarizes the quantitative average results for BUSI dataset, it also includes the standard deviation for each metric. To facilitate analysis, the results are grouped into benign and malignant masses.

**Figure 4:**
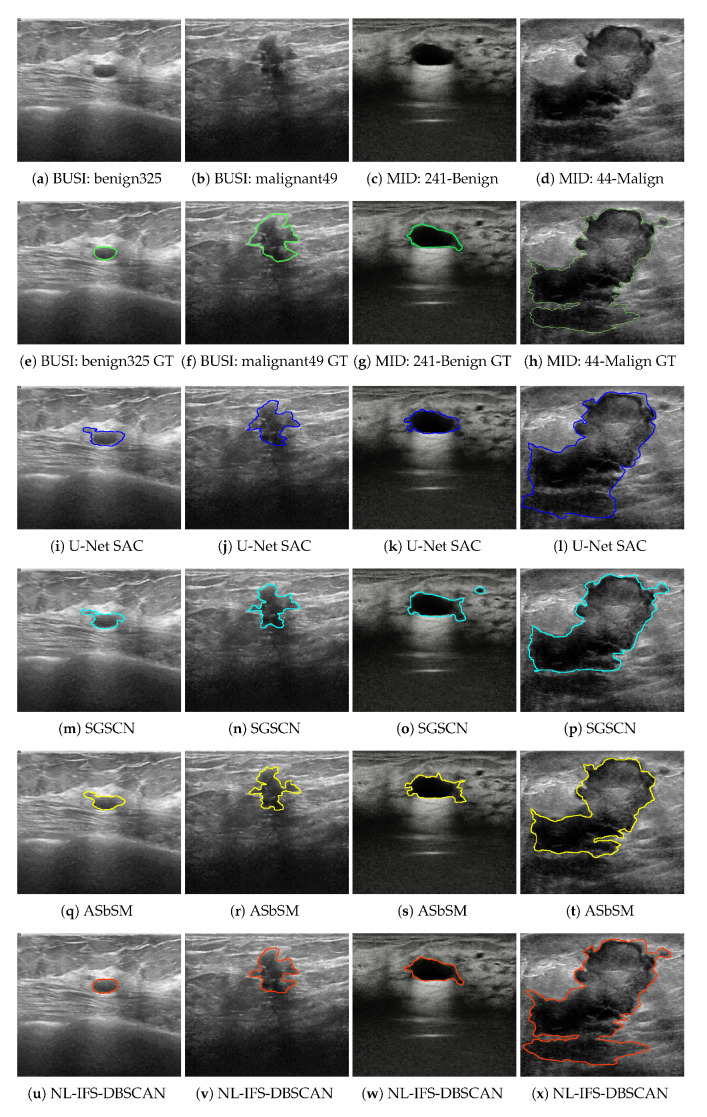
Sample segmentation results on the considered databases.

For the JSC and DM metrics, a value close to unity suggests better segmentation, while for HD and MCR, a minimum value represents the best performance. In considering this remark, the current proposal had the best efficiency for both breast mass types. In each specific case, JSC=0.907, DM=0.913, HD=7.025, and MCR=6.431 for benign masses, and JSC=0.897, DM=0.900, HD=8.666, and MCR=8.016 for malignant masses. It is worth noting that the current proposal had the lowest standard deviation. In general terms, it can be mentioned that after the introduced method, the performance order was the ASbSM, U-Net SAC, and SGSCN algorithms. Figure 4 presents a sample image pair of each database, their ground truths, and the segmentation developed by each evaluated algorithm. As shown in Figure 4u,v, the best tumor delimitation was provided by the proposed method, which strongly supports the quantitative results. It can be seen that the comparative methods achieved a good segmentation; however, they differ a little with respect to the manual segmentation.

### 4.3. Experiment 2: MID Dataset

Using the same metrics in the previous experiment, all algorithms and methods were evaluated by using MID dataset. In Table 2, one can see the numerical results provided by each approach; it is evident that all methods had a light performance decrease with respect to the first dataset. Nevertheless, the proposed method maintained its superiority in efficiency terms. Standing out were specific values, such as JSC=0.890, DM=0.905, HD=8.370 and MCR=7.241 for benign masses, as well as JSC=0.881, DM=0.898, HD=8.865 and MCR=7.808 for malignant masses. Special attention should be given to the fact that the standard deviation increased its value; in other words, this means that the processing of the second dataset was more challenging than that of the first. Furthermore, with respect to visual results, Figure 4c,d depict samples for benign and malignant masses, while Figure 4g,h depct their respective ground truth. The complexity of the regions of interest can be observed, which made it difficult for comparative methods to carry out an appropriate delimitation. In contrast, the proposed method achieved a better approximation to manual segmentation.

Both experiments demonstrated that the current proposals had remarkable performance. There are, however, aspects that should be addressed to improve the efficiency. For example, all the processing was carried considering the pixel intensity as one feature, but perhaps it might be worth considering the possibility of using texture, spatial position, edges, or more sophisticated features. It is a highly desirable non-parametric system, but the Intuitionistic Fuzzy clustering algorithm is inherently defined with respect to some parameters. Although three values were suggested, in real situations where the masses are very small, it may be necessary to increase the granularity of the superpixels and thus modify those parameters. To solve this issue, perhaps some heuristics can be considered to perform the auto-tuning.

## 5. Conclusions

This study proposes the use of a traditional image processing approach for breast ultrasound lesion delimitation, consisting of a preprocessing step and two clustering procedures. First, the multiplicative noise was reduced by the very popular Nonlocal Means filter. Then, a local region segmentation was developed by Intuitionistic Fuzzy Clustering, which allowed us to obtain small regions so-called superpixels with high adherence to the local boundaries. At a later time, those superpixels where clustered with the aid of DBSCAN, in order to develop a global tumor delimitation.

The effectiveness of our proposal was verified by quantitative metrics such as the Jaccard Similarity Coefficient, Dice Measure, Hausdorff Distance, and the Misclassification Ratio and compared with some reference methods taken from the state of the art. The experimentation was performed using the BUSI and MID databases, since both were freely accessed and had benign and malignant masses. The average results revealed that our proposal had superior performance with respect to all evaluated comparative state-of-art methods, since the delimitation was more similar to the one supplied in the ground-truth, for two types of tumors.

There are, however, limitations that should be addressed to improve the efficiency, since at this moment it is only working with the pixel intensities. Future work will consider enhancing the proposed system by means of better features, e.g., region analysis, texture analysis, and pixel and image statistics. Another aspect that will be considered will dispense with the preprocessing stage and enhance the clustering algorithms via robust estimation, in order to make them noise-tolerant.

## Figures and Tables

**Table 1:** Quantitative results for the BUSI dataset.

Algorithm	Masses	*JSC*	*DM*	*HD*	*MCR*
SAC U-Net	Benign	0.871 ± 0.073	0.896 ± 0.083	7.342 ± 1.801	7.029 ± 1.253
SGSCN	Benign	0.864 ± 0.108	0.878 ± 0.110	8.290 ± 2.347	8.590 ± 2.032
ASbSM	Benign	0.892 ± 0.088	0.905 ± 0.085	7.756 ± 2.103	7.167 ± 1.747
NL-IFS-DBSCAN	Benign	0.907 ± 0.064	0.913 ± 0.083	7.025 ± 1.274	6.431 ± 0.913
SAC U-Net	Malignant	0.848 ± 0.089	0.866 ± 0.092	10.160 ± 3.192	9.033 ± 2.418
SGSCN	Malignant	0.831 ± 0.127	0.859 ± 0.152	11.048 ± 3.211	10.065 ± 2.841
ASbSM	Malignant	0.851 ± 0.112	0.872 ± 0.108	9.139 ± 2.879	8.693 ± 2.032
NL-IFS-DBSCAN	Malignant	0.879 ± 0.082	0.900 ± 0.087	8.666 ± 1.545	8.016 ± 1.268

**Table 2:** Quantitative results for MID dataset.

Algorithm	Masses	*JSC*	*DM*	*HD*	*MCR*
SAC U-Net	Benign	0.847 ± 0.086	0.883 ± 0.098	9.822 ± 2.120	8.262 ± 1.580
SGSCN	Benign	0.817 ± 0.136	0.853 ± 0.119	10.983 ± 2.986	9.041 ± 2.963
ASbSM	Benign	0.870 ± 0.115	0.882 ± 0.107	9.139 ± 2.654	7.797 ± 2.121
NL-IFS-DBSCAN	Benign	0.890 ± 0.071	0.905 ± 0.081	8.370 ± 1.663	7.241 ± 1.240
SAC U-Net	Malignant	0.791 ± 0.109	0.834 ± 0.090	10.882 ± 2.011	8.573 ± 1.805
SGSCN	Malignant	0.753 ± 0.172	0.808 ± 0.157	11.292 ± 2.693	9.743 ± 2.759
ASbSM	Malignant	0.818 ± 0.148	0.869 ± 0.139	9.946 ± 2.129	8.428 ± 1.792
NL-IFS-DBSCAN	Malignant	0.881 ± 0.080	0.898 ± 0.073	8.865 ± 1.799	7.808 ± 1.441

## Data Availability

Not applicable.
